# A Novel PARAFAC Model for Processing the Nested Vector-Sensor Array

**DOI:** 10.3390/s18113708

**Published:** 2018-10-31

**Authors:** Wei Rao, Dan Li, Jian Qiu Zhang

**Affiliations:** 1 Key Laboratory for Information Science of Electromagnetic Waves (MoE), School of Information Science and Technology, Fudan University, Shanghai 200433, China; lidan@fudan.edu.cn (D.L.); jqzhang01@fudan.edu.cn (J.Q.Z.); 2 Nanchang Institute of Technology, Nanchang 330099, China

**Keywords:** direction of arrival estimation, nested array, vector sensor, parallel factor (PARAFAC) decomposition

## Abstract

In this paper, a novel parallel factor (PARAFAC) model for processing the nested vector-sensor array is proposed. It is first shown that a nested vector-sensor array can be divided into multiple nested scalar-sensor subarrays. By means of the autocorrelation matrices of the measurements of these subarrays and the cross-correlation matrices among them, it is then demonstrated that these subarrays can be transformed into virtual scalar-sensor uniform linear arrays (ULAs). When the measurement matrices of these scalar-sensor ULAs are combined to form a third-order tensor, a novel PARAFAC model is obtained, which corresponds to a longer vector-sensor ULA and includes all of the measurements of the difference co-array constructed from the original nested vector-sensor array. Analyses show that the proposed PARAFAC model can fully use all of the measurements of the difference co-array, instead of its partial measurements as the reported models do in literature. It implies that all of the measurements of the difference co-array can be fully exploited to do the 2-D direction of arrival (DOA) and polarization parameter estimation effectively by a PARAFAC decomposition method so that both the better estimation performance and slightly improved identifiability are achieved. Simulation results confirm the efficiency of the proposed model.

## 1. Introduction

The vector sensors, e.g., the acoustic [1] and electromagnetic (EM) [2] ones, can record two to six signal components on a collocated sensor. Hence, the redundancy of signals is one of their advantages. By means of a single polarized vector sensor, Yuan in [3] achieved estimating the direction of arrival (DOA) and the polarization of a completely-polarized polynomial-phase signal of an arbitrary degree. In [4,5], the vector sensors were applied to the target localization. A multiple-input multiple-output (MIMO) array system with the EM vector antennas was presented in [6]. However, all these contributions utilized the so-called “long-vector” approach which could destroy the multidimensional structure of the received signals of vector sensors [7]. In order to fully utilize the multidimensional structure information of vector sensors, a tensor decomposition method for effectively estimating vector-sensor-based signal parameters was proposed in [8]. 

The topic of source localization with fewer sensors than sources has received extensive attention in recent years. One of the most effective methods for doing that is to construct a virtual array, i.e., the difference co-array from the physical array covariance, with a higher degrees-of-freedom (DOF) than that of the physical array. One of the typical schemes reported in literature is the nested array [9]. The nested array can achieve *O*(*N*^2^) DOF with *N* sensors when two or more uniform linear arrays (ULAs) with increasing inter-element spacing are suitably combined to form a difference co-array. The nested array theory has been applied to various scenarios, e.g., the off-grid DOA estimations [10], two-dimensional arrays [11], conformal arrays [12], L-shaped nested arrays [13,14], adaptive beamforming [15], wideband signals [16], distributed sources [17], and spatial-temporal nested sampling [18].

For utilizing the 1-D array to achieve the 2-D DOA estimation and resolve significantly more sources than the actual number of physical sensors, the nested vector-sensor array was proposed in [19]. Since the data structure of the nested vector-sensor array is more complex, the multilinear algebra, that is the tensor algebra [20], was utilized in [19]. Although the measurements of the nested vector-sensor array in [19] were modeled as a tensor, the ones of the difference co-array constructed from the nested vector-sensor array were described as a matrix. As a result, 1/*M* (*M* is the components number in a vector sensor) observation data of the difference co-array were only exploited, which means that the redundancy of signals offered by the vector sensors has not been taken full advantage. Furthermore, for achieving the 2-D DOA and polarization parameter estimation, it has to apply at least two-way spectral peak searching, maybe up to four-way, to the difference co-array covariance tensor as shown in [19], which implies that the high computational complexity has to be paid. 

In order to take full advantage of the redundancy of signals offered by the nested vector-sensor array and avoid the multidimensional spectrum peak search, a novel parallel factor (PARAFAC) model for processing such an array is proposed in this paper. Analyses show that by dividing the measurement tenor of the nested vector-sensor array into matrices, we can obtain *M* matrix models corresponding to *M* independent nested scalar-sensor subarrays, each of which is constructed from the components of the *N* vector sensors with the same orientation. From the autocorrelation matrices of the received signals of the *M* subarrays and cross-correlation matrices among them, *M* measurement matrices corresponding to *M* virtual ULAs with $N^{2}/2+N-1$ scalar sensors are obtained. Since these virtual scalar-sensor ULAs enjoy the same spatial and equivalent temporal diversity spaces, we can combine them to form a new virtual ULA with $N^{2}/2+N-1$ vector sensors and *M* snapshots, and model it as a tensor with a PARAFAC decomposition form. In this way, all of the measurements from the difference co-array of the original nested vector-sensor array are described as a PARAFAC model, instead of a matrix one reported in [19]. It also means that these measurements are fully exploited to improve the estimation performance and the identifiability of the difference co-array when a PARAFAC decomposition method is applied to our model. Simulation results confirm the correctness of the analytical results and verify the effectiveness of the proposed model.

The reminders of the paper are organized as follows. In Section 2, three tensor operators required by this paper are simply reviewed. The PARAFAC model with an explicit diversity structure for a nested vector-sensor array is given in Section 3. In Section 4, the novel PARAFAC model for the difference co-array of the nested vector-sensor array is proposed. How to employ the proposed model to achieve the source localization and polarization estimation is reported in Section 5. Simulation results are presented in Section 6. Section 7 concludes this paper.

Notations: ${\left(\cdot \right)}^{*}$, ${\left(\cdot \right)}^{T}$, ∘, ⊗, and ⊙ denote conjugation, transpose, outer product, Kronecker product, and Khatri-Rao product, respectively.

## 2. Tensor Algebra Prerequisites

For the readers’ convenience, some most relevant tensor operations are reviewed here. For a complete introduction to them, please refer to [20,21,22].

**Definition** **1.** *(The PARAFAC decomposition): Let*$A\in {\mathbb{C}}^{I_{1}\times \cdots \times I_{N}}$*be a Nth-order tensor, then the PARAFAC decomposition of*A*is a weighted sum of rank-1 tensors, defined as*

(1) $$A=\sum_{k=1}^{K}c_{k}a_{k}^{\left(1\right)}\circ a_{k}^{\left(2\right)}\circ \cdots \circ a_{k}^{\left(N\right)}\;$$

*where*$c_{k}$*is a constant coefficient,*$a_{k}^{\left(n\right)}$*is a vector of size*$I_{n}$*(*n=1,2,…,N).

*The so-called “factor matrices” of the decomposition*$A^{\left(n\right)}\in {\mathbb{C}}^{I_{n}\times K}$*for*n=1,2,…,N*are written as*$A^{\left(n\right)}=[a_{1}^{\left(n\right)},\ldots ,a_{K}^{\left(n\right)}]$.

**Definition** **2.** *(The tensor contraction): Given* $A\in {\mathbb{C}}^{I_{1}\times \cdots \times I_{N}}$ *,* $B\in {\mathbb{C}}^{J_{1}\times \cdots \times J_{M}}$ *,* 1≤p≤N *,* 1≤q≤M *, and* $I_{p}=I_{q}$ *, then the contraction between* A *and* B *in the pth and qth modes is denoted by* $C={\langle A,B\rangle }_{\left(p,q\right)}\in {\mathbb{C}}^{I_{1}\times \cdots \times I_{p-1}\times I_{p+1}\times \cdots \times I_{N}\times J_{1}\cdots \times J_{q-1}\times J_{q+1}\times \cdots \times J_{M}}$ *with its element as*

(2) $$c_{i_{1},\ldots ,i_{p-1},i_{p+1},\ldots ,i_{N},j_{1},\ldots ,j_{q-1},j_{q+1},\ldots ,j_{M}}=\sum_{i_{p}=1}^{I_{p}}a_{i_{1},\ldots ,i_{N}}b_{j_{1},\ldots ,j_{M}}\;$$

**Definition** **3.** *(The matricization of the PARAFAC decomposition): For a*N*th-order PARAFAC model*$A=\sum_{k=1}^{K}c_{k}a_{k}^{\left(1\right)}\circ \cdots \circ a_{k}^{\left(N\right)}\in {\mathbb{C}}^{I_{1}\times \cdots \times I_{N}}$*where*$c_{k}$*is a constant coefficient and*$a_{k}^{\left(n\right)}\in {\mathbb{C}}^{I_{n}}$*(*n=1,2,…,N*), let the ordered sets*$A=\{a_{1},\ldots ,a_{L}\}$*and*$B=\{b_{1},\ldots ,b_{M}\}$*be a partitioning of the dimensions*{1,…,N}*, then the matricization of*A*, denoted by*$A_{A,B}\in {\mathbb{C}}^{N_{1}\times N_{2}}$*with*$N_{1}=\prod_{n\in A}I_{n}$*and*$N_{2}=\prod_{n\in B}I_{n}$, *is defined as*

(3) $$A_{A,B}=\sum_{k=1}^{K}c_{k}b_{k}^{\left(1\right)}\circ b_{k}^{\left(2\right)}\in {\mathbb{C}}^{N_{1}\times N_{2}}\;$$

*where*$b_{k}^{\left(1\right)}=a^{\left(a_{L}\right)}⊗a^{\left(a_{L-1}\right)}⊗\cdots ⊗a^{\left(a_{1}\right)}$*and*$b_{k}^{\left(2\right)}=a^{\left(b_{M}\right)}⊗a^{\left(b_{M-1}\right)}⊗\cdots ⊗a^{\left(b_{1}\right)}$.

## 3. Tensor Model for a Nested Vector-Sensor Array

In this section, we will arrange the measurements and noise from all of the components of the sensors in the nested vector-sensor array into a third-order tensor. 

**Figure 1:**
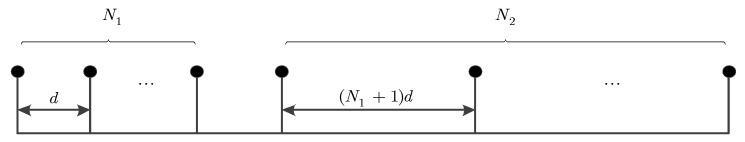
A 2-level nested vector-sensor array.

As shown in Figure 1, a 2-level nested vector-sensor array containing $N_{1}$ vector-sensors in the inner ULA and $N_{2}$ vector sensors in the outer ULA is taken into consideration. Without loss of generality, assume that all of the vector sensors in the array are located along *z*-axis, the total number of sensors $N=N_{1}+N_{2}$ is even, $N_{1}=N_{2}=N/2$, the inter-sensor spacing in the inner ULA is one half of the signal wavelength, i.e., d=λ/2, and the one in the outer ULA is $(N_{1}+1)d$. Each vector sensor contains *M* components. There are *K* narrowband far-field uncorrelated signals impinge on the array from the distinct directions with elevation and azimuth angles ${\left\{(\theta_{k},\phi_{k})\right\}}_{k=1}^{K}$, where $\theta_{k}\in [-\pi /2,\pi /2]$ and $\phi_{k}\in [-\pi ,\pi )$. Let $u_{k}={\left[\cos(\theta_{k})\cos(\phi_{k}),\cos(\theta_{k})\sin(\phi_{k}),\sin(\theta_{k})\right]}^{T}$ be the direction cosine of the *k*th source, and $r_{n}={\left[x_{n},y_{n},z_{n}\right]}^{T}$ be the position vector of the nth sensor. In this way, the measurement matrix of the array at time *t* is given as [19]

(4) $$Y(t)=\sum_{k=1}^{K}\left(d_{k}\circ p_{k})x_{k}(t\right)+E(t)\in {\mathbb{C}}^{N\times M},\;1\leq t\leq T,$$

where $d_{k}={\left[e^{j2\pi u_{k}^{T}r_{1}/\lambda }\;,\ldots ,\;e^{j2\pi u_{k}^{T}r_{N}/\lambda }\right]}^{T}$ is the spatial steering vector, $x_{k}(t)$ is the *k*th source signal, E(t) is the corresponding noise matrix, *T* (≥K) is the number of snapshots, and $p_{k}\in {\mathbb{C}}^{M}$ is the spatial response vector of the vector sensor located at the origin. Note that for the acoustic vector sensors [1] M=4 and $p_{k}=[1,u_{k}^{T}{]}^{T}$, while for the electromagnetic vector sensors [2] M=6 and

$$p_{k}=\left[\begin{array}{cc} -\sin(\phi_{k}) & -\cos(\phi_{k})\sin(\theta_{k})\\ \cos(\phi_{k}) & -\sin(\phi_{k})\sin(\theta_{k})\\ 0 & \cos(\theta_{k})\\ -\cos(\phi_{k})\sin(\theta_{k}) & \sin(\phi_{k})\\ -\sin(\phi_{k})\sin(\theta_{k}) & -\cos(\phi_{k})\\ \cos(\theta_{k}) & 0 \end{array}\right]\left[\begin{array}{c} \cos(\gamma_{k})\\ \sin(\gamma_{k})e^{j\eta_{k}} \end{array}\right],$$

where $\gamma_{k}\in [0,2\pi ]$ and $\eta_{k}\in (-\pi ,\pi ]$ are the polarization auxiliary and phase difference angles of the source, respectively.

Based on Equation (4), let $Y(t)\in {\mathbb{C}}^{N\times M}$ (1≤t≤T) be the *t*th frontal slice [20] of the tensor $Y\in {\mathbb{C}}^{N\times M\times T}$, then Y can be expressed as

(5) $$Y=\sum_{k=1}^{K}d_{k}\circ p_{k}\circ x_{k}+E\in {\mathbb{C}}^{N\times M\times T}\;$$

where $x_{k}={\left[x_{k}(1),\ldots ,x_{k}(T)\right]}^{T}$ is the *k*th signal vector, and $E\in {\mathbb{C}}^{N\times M\times T}$ is the corresponding noise tensor whose *t*th frontal slice is $E(t)\in {\mathbb{C}}^{N\times M}$. Obviously, this tensor model is of an explicit diversity structure. More precisely, the three dimensions of Y are respectively corresponding to the spatial, polarized, and temporal diversity spaces of a nested vector-sensor array. Comparing the signal model of Equation (10) in [19] with Equation (5) for the same nested vector-sensor array, one can see that unlike the matrix model in [19] the tensor model Y in Equation (5) contains all the measurements of the array, and more importantly it is of a natural PARAFAC decomposition form as Equation (1). It means that the PARAFAC decomposition can be applied to Y for estimating the $d_{k}$, $p_{k}$, and $x_{k}$ if required.

## 4. New Model for the Difference Co-Array

Similar to Equation (5), here a PARAFAC model for the difference co-array constructed from the array covariance of the nested vector-sensor array will be given. 

Let $Y^{\left(m\right)}$ and $E^{\left(m\right)}$ be the *m*th lateral slices of Y and E [20], respectively, then $Y^{\left(m\right)}$ can be given by

(6) $$Y^{\left(m\right)}=\sum_{k=1}^{K}(d_{k}\circ x_{k})p_{k}^{\left(m\right)}+E^{\left(m\right)}\in {\mathbb{C}}^{N\times T},\;1\leq m\leq M,$$

where $p_{k}^{\left(m\right)}$ is the mth entry of $p_{k}$.

Comparing Equation (5) with Equation (6), one can find that $Y^{\left(m\right)}$ for 1≤m≤M can be viewed as the measurement matrices of *M* independent nested scalar-sensor subarrays, each of which is constructed from the components of the *N* vector sensors with the same orientation.

Utilizing the Definition 2, one can get the second-order automoments, i.e., autocorrelation matrices, of the *M* nested scalar-sensor subarrays as:

(7) $$\begin{array}{cc} R(m,m) & =E\left[Y^{\left(m\right)}\circ Y^{\left(m\right)}{}^{*}\right]\\ & \approx \frac{1}{T}{\langle Y^{\left(m\right)}\circ Y^{\left(m\right)}{}^{*}\rangle }_{\left(2,2\right)}\\ & =\sum_{k=1}^{K}(d_{k}\circ d_{k}^{*})p_{k}^{\left(m\right)}p_{k}^{\left(m\right)}{}^{*}\sigma_{k}^{2}+\sigma_{e}^{2}I \end{array},\;1\leq m\leq M,$$

where $\sigma_{k}^{2}$ and $\sigma_{e}^{2}$ are the signal and noise powers, respectively, and I is the M×M identity matrix.

Similarly, the second-order cross-moments, i.e., cross-correlation matrices, among the *m*th subarray and the others are given as:

(8) $$\begin{array}{cc} R(m,l) & =E\left[Y^{\left(m\right)}\circ Y^{\left(l\right)}{}^{*}\right]\\ & \approx \frac{1}{T}{\langle Y^{\left(m\right)}\circ Y^{\left(l\right)}{}^{*}\rangle }_{\left(2,2\right)}\\ & =\sum_{k=1}^{K}(d_{k}\circ d_{k}^{*})p_{k}^{\left(m\right)}p_{k}^{\left(l\right)}{}^{*}\sigma_{k}^{2} \end{array},\;1\leq m,l\leq M,\;m\neq l\;$$

Based on Equations (7) and (8), let R(m,l) be the lth (1≤l≤M) frontal slice of a tensor $R^{\left(m\right)}\in {\mathbb{C}}^{N\times N\times M}$, then $R^{\left(m\right)}$ can be written as:

(9) $$R^{\left(m\right)}=\sum_{k=1}^{K}(d_{k}\circ d_{k}^{*}\circ p_{k}^{*})p_{k}^{\left(m\right)}\sigma_{k}^{2}+\sigma_{e}^{2}I^{\left(m\right)},\;1\leq m\leq M$$

where $I^{\left(m\right)}\in {\mathbb{C}}^{N\times N\times M}$ with the elements

$$i_{j_{1},j_{2},j_{3}}^{\left(m\right)}=\{\begin{array}{cc} 1 & if\;j_{1}=j_{2}\;\mathrm{and}\;j_{3}=m\\ 0 & else \end{array}.$$

Applying the Definition 1 with A={1,2} and B={3} to $R^{\left(m\right)}$ we have

(10) $$R_{\left\{1,2\}\{3\right\}}^{\left(m\right)}=\sum_{k=1}^{K}(a_{k}\circ p_{k}^{*})p_{k}^{\left(m\right)}\sigma_{k}^{2}+\sigma_{e}^{2}I_{\left\{1,2\}\{3\right\}}^{\left(m\right)},\;1\leq m\leq M,$$

where $a_{k}=d_{k}^{*}⊗d_{k}$. Note that $d_{k}$ is the spatial steering vector of the original nested array with *N* sensors, hence by removing the repeated rows and sorting the remaining ones in $a_{k}$, one can obtain the spatial steering vector of a virtual ULA with $N^{2}/2+N-1$ sensors [9]. Similarly, according to $a_{k}$ in Equation (10) we remove the repeated rows and sort the remaining ones in $R_{\left\{1,2\}\{3\right\}}^{\left(m\right)}$, then we have:

(11) $${\bar{R}}_{\left\{1,2\}\{3\right\}}^{\left(m\right)}=\sum_{k=1}^{K}({\bar{a}}_{k}\circ p_{k}^{*})p_{k}^{\left(m\right)}\sigma_{k}^{2}+\sigma_{e}^{2}{\bar{I}}_{\left\{1,2\}\{3\right\}}^{\left(m\right)},\;1\leq m\leq M,$$

where:

(12) $${\bar{a}}_{k}={\left[e^{-j(N^{2}/4+N/2-1)\pi \sin(\theta_{k})},\ldots \;,e^{-j\pi \sin(\theta_{k})},1,e^{j\pi \sin(\theta_{k})},\ldots ,e^{j(N^{2}/4+N/2-1)\pi \sin(\theta_{k})}\right]}^{T}.$$

Let ${\bar{R}}_{\left\{1,2\}\{3\right\}}^{\left(m\right)}={Y^{\prime }}^{\left(m\right)}$, $p_{k}^{*}\sigma_{k}^{2}=x_{k}^{\prime }$ and $\sigma_{e}^{2}{\bar{I}}_{\left\{1,2\}\{3\right\}}^{\left(m\right)}={E^{\prime }}^{\left(m\right)}$, then ${\bar{R}}_{\left\{1,2\}\{3\right\}}^{\left(m\right)}$ can be rewritten as

(13) $${Y^{\prime }}^{\left(m\right)}=\sum_{k=1}^{K}({\bar{a}}_{k}\circ x_{k}^{\prime })p_{k}^{\left(m\right)}+{E^{\prime }}^{\left(m\right)}\in {\mathbb{C}}^{(N^{2}/2+N-1)\times M},\;1\leq m\leq M.$$

Comparing Equation (6) with Equation (13), one can see that if $x_{k}^{\prime }$ is taken as the equivalent signal vector, then ${Y^{\prime }}^{\left(m\right)}$ can be viewed as the measurement matrix of a virtual ULA with $N^{2}/2+N-1$ scalar sensors. Note that ${\bar{R}}_{\left\{1,2\}\{3\right\}}^{\left(m\right)}$ contains *M* equivalent snapshots. Hence, from the *M* nested subarrays with *N* scalar sensors as shown in Equation (6), one can construct *M* virtual ULAs with $N^{2}/2+N-1$ scalar sensors as shown in Equation (13). 

Since the *M* scalar-sensor ULAs contain the same spatial (${\bar{a}}_{k}$) and equivalent temporal ($x_{k}^{\prime }$) diversity spaces as shown in Equation (13), ${Y^{\prime }}^{\left(m\right)}$ for 1≤m≤M can be arranged into a three-way tensor denoted by $Y^{\prime }$, where ${Y^{\prime }}^{\left(m\right)}$ is the mth (1≤m≤M) lateral slices of $Y^{\prime }$. Let ${E^{\prime }}^{\left(m\right)}$ be the mth (1≤m≤M) lateral slices of $E^{\prime }$, then $Y^{\prime }$ can be expressed as

(14) $$Y^{\prime }=\sum_{k=1}^{K}{\bar{a}}_{k}\circ p_{k}\circ x_{k}^{\prime }+E^{\prime }\in {\mathbb{C}}^{(N^{2}/2+N-1)\times M\times M}.$$

Comparing Equation (14) with Equation (5), one can find that the proposed PARAFAC model $Y^{\prime }$ corresponds to a virtual ULA with $N^{2}/2+N-1$ vector sensors and M snapshots. From Equations (7) and (8), it is easy to verify that $Y^{\prime }$ consists of all the measurements of the difference co-array constructed from the original nested vector-sensor array. Hence, we call $Y^{\prime }$ in Equation (14) as the PARAFAC model for the difference co-array of a nested vector-sensor array, which will be used to improve the performance of the nested vector-sensor array.

**Remark** **1.** *As shown in Equation (14), a virtual ULA with* $N^{2}/2+N-1$ *vector sensors and M snapshots has been constructed from the original nested array with N vector sensors. It should be noted that such a virtual vector-sensor ULA can be considered as the complete difference co-array of the nested vector-sensor array, because* $Y^{\prime }$ *contains all of the data in the array covariance of the original nested array. In contrast, 1/M data, corresponding to a single snapshot, from the array covariance of the original nested array are only used for constructing the virtual vector-sensor ULA as given in [19]. Furthermore, the virtual vector-sensor ULA constructed by [19] was modeled as a matrix, whereas our virtual vector-sensor ULA is modeled as a tensor with a PARAFAC decomposition form. Based on the proposed model in Equation (14), a significant performance improvement can, hence, be expected.*

## 5. 2-D DOA and Polarization Parameter Estimation

### 5.1. Tensor-Based Spatial Smoothing

In Equation (14), $x_{k}^{\prime }=p_{k}^{*}\sigma_{k}^{2}$ can be viewed as the *k*th equivalent signal vector with *M* samples. Hence, before estimating 2-D DOAs and polarization parameters of sources, one can employ the tensor-based spatial smoothing technique [23] to increase the equivalent snapshots so that the identifiability of the virtual vector-sensor ULA can be increased. To this end, we divide the $(N^{2}/2+N-1)\times M$ matrix ${y^{\prime }}^{\left(m\right)}$ (13) into $N_{s}$ overlapping sub-matrices of size $N_{0}=N^{2}/2+N-N_{s}$, where the $n_{s}$th ($1\leq n_{s}\leq N_{s}$) sub-matrix corresponding to the $n_{s}$th to ($n_{s}+N_{0}-1$)th rows of ${y^{\prime }}^{\left(m\right)}$ is expressed as:

(15) $${Y^{\prime }}^{\left(m,n_{s}\right)}=\sum_{k=1}^{K}({\bar{a}}_{k,n_{s}}\circ x_{k}^{\prime })p_{k}^{\left(m\right)}+{E^{\prime }}^{\left(m,n_{s}\right)}\in {\mathbb{C}}^{N_{0}\times M},\;1\leq m\leq M,\;1\leq n_{s}\leq N_{s},$$

where ${\bar{a}}_{k,n_{s}}$ is corresponding to the $n_{s}$th to $\left(n_{s}+N_{0}-1\right)$th elements of ${\bar{a}}_{k}$. Since ${\bar{a}}_{k}$ is the spatial steering vector of the virtual ULA expressed as (12), one can denote ${\bar{a}}_{k,n_{s}}$ as:

(16) $${\bar{a}}_{k,n_{s}}={\bar{a}}_{k,1}e^{j(n_{s}-1)\pi \sin(\theta_{k})},\;1\leq n_{s}\leq N_{s},$$

where ${\bar{a}}_{k,1}$ is corresponding to the first to $N_{0}$th elements of ${\bar{a}}_{k}$.

Now, we rewrite ${Y^{\prime }}^{\left(m,n_{s}\right)}$ as:

(17) $${Y^{\prime }}^{\left(m,n_{s}\right)}=\sum_{k=1}^{K}({\bar{a}}_{k,1}\circ x_{k}^{\prime })e^{j(n_{s}-1)\pi \sin(\theta_{k})}p_{k}^{\left(m\right)}+{E^{\prime }}^{\left(m,n_{s}\right)}\in {\mathbb{C}}^{N_{0}\times M}.$$

Let ${Y^{\prime }}^{\left(m,n_{s}\right)}$ and ${E^{\prime }}^{\left(m,{\bar{n}}_{s}\right)}$ be the $n_{s}$th ($1\leq n_{s}\leq N_{s}$) frontal slices of the $N_{0}\times M\times N_{s}$ tensors $Z^{\left(m\right)}$ and $W^{\left(m\right)}$, respectively, then $Z^{\left(m\right)}$ can be given by

(18) $$Z^{\left(m\right)}=\sum_{k=1}^{K}({\bar{a}}_{k,1}\circ x_{k}^{\prime }\circ b_{k})p_{k}^{\left(m\right)}+W^{\left(m\right)}\in {\mathbb{C}}^{N_{0}\times M\times N_{s}},\;1\leq m\leq M,$$

where $b_{k}={\left[1,e^{j\pi \sin(\theta_{k})},\ldots \;,e^{j(N_{s}-1)\pi \sin(\theta_{k})}\right]}^{T}$.

Applying the Definition 3 with A={1} and B={2,3} to $Z^{\left(m\right)}$ we have

(19) $$Z_{\left\{1\}\{2,3\right\}}^{\left(m\right)}=\sum_{k=1}^{K}({\bar{a}}_{k,1}\circ s_{k})p_{k}^{\left(m\right)}+W_{\left\{1\}\{2,3\right\}}^{\left(m\right)}\in {\mathbb{C}}^{N_{0}\times N_{s}M},\;1\leq m\leq M,$$

where $s_{k}=b_{k}⊗x_{k}^{\prime }\in {\mathbb{C}}^{N_{s}M}$.

Note that $Z_{\left\{1\}\{2,3\right\}}^{\left(m\right)}$ for 1≤m≤M can be arranged into a three-way tensor denoted by $Z\in {\mathbb{C}}^{N_{0}\times M\times N_{s}M}$. Assuming $Z\in {\mathbb{C}}^{N_{0}\times M\times N_{s}M}$ and $W\in {\mathbb{C}}^{N_{0}\times M\times N_{s}M}$ whose *m*th (1≤m≤M) lateral slices are $Z_{\left\{1\}\{2,3\right\}}^{\left(m\right)}$ and $W_{\left\{1\}\{2,3\right\}}^{\left(m\right)}$, respectively, we have

(20) $$Z=\sum_{k=1}^{K}{\bar{a}}_{k,1}\circ p_{k}\circ s_{k}+W\in {\mathbb{C}}^{N_{0}\times M\times N_{s}M}.$$

Comparing Equation (14) with Equation (20), one can see that a longer ULA with $N^{2}/2+N-1$ vector sensors and *M* snapshots is transformed into the short one with $N_{0}(=N^{2}/2+N-N_{s})$ vector sensors and $N_{s}M$ snapshots. Its purpose is to overcome the problems that may occur when the source matrix is rank deficient. Hence, similar to the traditional spatial smoothing technique, the goal of this way here is to obtain a new full rank source signal matrix at the expense of a reduced effective aperture.

### 5.2. Uniqueness

In Equation (20) $s_{k}=b_{k}⊗x_{k}^{\prime }\in {\mathbb{C}}^{N_{s}M}$ is the *k*th equivalent signal vector with $N_{s}M$ samples, and its corresponding factor matrix (i.e., the new source signal matrix) is:

(21) $$S=[s_{1},\ldots ,s_{K}]\in {\mathbb{C}}^{N_{s}M\times K}.$$

Let ${\bar{A}}_{1}=[{\bar{a}}_{1,1},\ldots ,{\bar{a}}_{K,1}]\in {\mathbb{C}}^{N_{0}\times K}$, $P=[p_{1},\ldots ,p_{K}]\in {\mathbb{C}}^{M\times K}$, $X^{\prime }=[x_{1}^{\prime },\ldots ,x_{K}^{\prime }]\in {\mathbb{C}}^{M\times K}$, and $B=[b_{1},\ldots ,b_{K}]\in {\mathbb{C}}^{N_{s}\times K}$ be the factor matrices corresponding to ${\bar{a}}_{k,1}$, $p_{K}$, $x^{\prime }$ and $b_{k}$, respectively. From $s_{k}=b_{k}⊗x_{k}^{\prime }$ we have:

(22) $$S=B⊙X^{\prime }\in {\mathbb{C}}^{N_{s}M\times K}.$$

Similar to [24,25,26], we also assume here that the *K* sources are uncorrelated and the source DOA pairs are restricted to satisfy the condition given by Theorem 4 in [12] so that the signal matrix $X=[x_{1},\ldots ,x_{K}]\in {\mathbb{C}}^{T\times K}$ (T≥K) with a full column rank is guaranteed. Under these assumptions and considering the Vandermonde structures of ${\bar{A}}_{1}$ and B, we have $k({\bar{A}}_{1})=\min(N_{0},K)$ and $k(B)=\min(N_{s},K)$, where k(A) represents the Kruskal rank [20] of the matrix A, which equals to the largest integer such that any set of k(A) columns of A is linearly independent.

Since $x_{k}^{\prime }=p_{k}^{*}\sigma_{k}^{2}$ and its corresponding factor matrix is $X^{\prime }=[x_{1}^{\prime },\ldots ,x_{K}^{\prime }]$, we can rewrite $X^{\prime }$ as:

(23) $$X^{\prime }=P^{*}\left[\begin{array}{ccc} \sigma_{1}^{2} & & \\ & \ddots & \\ & & \sigma_{K}^{2} \end{array}\right].$$

Obviously, the Kruskal rank of $X^{\prime }$ is depending on the $P^{*}$ (or P). So we have $k(X^{\prime })=k(P)\geq \min(4,K)$ in general for electromagnetic vector sensors [27] and $k(X^{\prime })=k(P)\geq \min(2,K)$ for acoustic vector sensors [28].

Using the Lemma 3.1 in [29], we have:

(24) $$k(S)=k(B⊙X^{\prime })\geq \min(k(B)+k(X^{\prime })-1,K)=\min(N_{s}+k(P)-1,K).$$

The PARAFAC model Z is essentially unique, if [20]

(25) $$k({\bar{A}}_{1})+k(P)+k(S)\geq 2K+2\Rightarrow k({\bar{A}}_{1})+k(S)\geq K+(K-k(P)+2).$$

According to Equation (25), for the case where $k({\bar{A}}_{1})=K$ (corresponding to $N_{0}\geq K$) and $k(S)=N_{s}+k(P)-1=K-k(P)+2$ (corresponding to $N_{s}+k(P)-1\leq K$), the uniqueness result of the proposed model can be given as follows

(26) $$K+k(P)+N_{s}+k(P)-1\geq 2K+2\Rightarrow K\leq N_{s}+2k(P)-3.$$

Taking $N_{0}\geq K$ into consideration and using Equation (26), we have

(27) $$2K\leq N_{s}+N_{0}+2k(P)-3.$$

Note that $N_{s}+N_{0}-1$ is equal to the vector sensor number of the longer vector-sensor ULA, i.e., $N^{2}/2+N-1$. Hence, Equation (27) can be rewritten as

(28) $$K\leq N^{2}/4+N/2+k(P)-1.5\Rightarrow K\leq N^{2}/4+N/2+k(P)-2.$$

Based on Equations (26) and (28), we have

(29) $$\left\{ \begin{array}{l} N_{s}=N^{2}/4+N/2-k(P)+1\\ N_{0}=N^{2}/2+N-N_{s}=N^{2}/4+N/2+k(P)-1 \end{array}\right..$$

Thus, applying the PARAFAC decomposition to the proposed Z under the conditions Equations (28) and (29), one can identify up to $N^{2}/4+N/2+2$ sources (corresponding to $N^{2}/4+N/2+3$ DOF) for the nested array with *N* electromagnetic vector sensors, and up to $N^{2}/4+N/2$ sources (corresponding to $N^{2}/4+N/2+1$ DOF) for the one with *N* acoustic vector sensors. In contrast, the method in [19] could resolve $N^{2}/4+N/2-1$ sources (corresponding to $N^{2}/4+N/2$ DOF) regardless of the electromagnetic vector sensors or acoustic ones. 

Utilizing the MATLAB function “cp3_alsls” provided by [30] to carry out the PARAFAC decomposition for Z, one can get the estimations ${\hat{\bar{A}}}_{1}$, $\hat{P}$ and $\hat{S}$. Then, the estimations $\left\{{\hat{\theta }}_{k}\right\}$ can be obtained from ${\hat{\bar{A}}}_{1}$, and $\left\{{\hat{\phi }}_{k}\right\}$, $\left\{{\hat{\gamma }}_{k}\right\}$, and $\left\{{\hat{\eta }}_{k}\right\}$ can be obtained from $\hat{P}$ as done in [8].

Moreover, from Equation (20), it can be seen that both the PARAFAC decomposition and the classical subspace methods can be employed to get the expected estimates. Let Z(t) ($1\leq t\leq N_{s}M$) be the *t*th frontal slice of Z, which can be viewed as the *t*th measurement matrix of the vector-sensor ULA, then the array covariance tensor is

(30) $$R=\frac{1}{N_{s}M}\sum_{t=1}^{N_{s}M}Z(t)\circ Z^{*}(t)\;$$

Based on Equation (29), one can apply the SORTE method [31] to detect the source number, and use the Tensor-MUSIC method described in detail in [19], to achieve 2-D DOA and polarization parameter estimation. In short, for the source localization and polarization estimation, the proposed model can be employed to do those with both the PARAFAC decomposition and Tensor-MUSIC. It should, however, be noted that, using the PARAFAC decomposition to do estimates, one cannot only avoid the multidimensional spectrum peak search but also improve the estimate performance because the data structure (e.g., Vandermonde) of Z is capitalized on enhancing the estimation accuracy further [23,32].

**Remark** **2.** *Compared to the method in [19], the identifiability of the proposed one is slightly improved. It should be emphasized that, since the proposed* Z *is a PARAFAC model, one can employ the PARAFAC decomposition to achieve the 2-D DOA and polarization parameter estimation. It implies that the 2-way spectral peak searching, maybe up to four-way when necessary, for the method in [19], can at least be avoided. Furthermore, the data structure (e.g., Vandermonde) in Z can be used by our model to improve the estimate performance further.*

### 5.3. Summary of the Proposed Method

The overall procedure of the proposed method is summarized in Table 1.

## 6. Numerical Examples

In this section, we will use numerical examples to show the effectiveness of our PARAFAC model and its analytical results. In all the simulations, except for the examples 6.5 and 6.6, the two-level nested array containing N=6 EM vector sensors, with $N_{1}=N_{2}=3$, is considered. Based on this nested vector-sensor array, we set $N_{0}=15$ and $N_{s}=9$ according to Equation (29) so that the proposed model can yield 15 DOF. In contrast, the model in [19] can yield 12 DOF. Moreover, the performance of a physical ULA with N=12 EM vector sensors whose DOF is 12 as well is taken as a benchmark. Notice that for the proposed model the PARAFAC decomposition is employed. For the model in [19] and the benchmark, the Tensor-MUSIC is used, where the angular resolution is fixed to $0.01^{{}^{\circ}}$.

### 6.1. Identifiability of the Proposed Model

**Figure 2:**
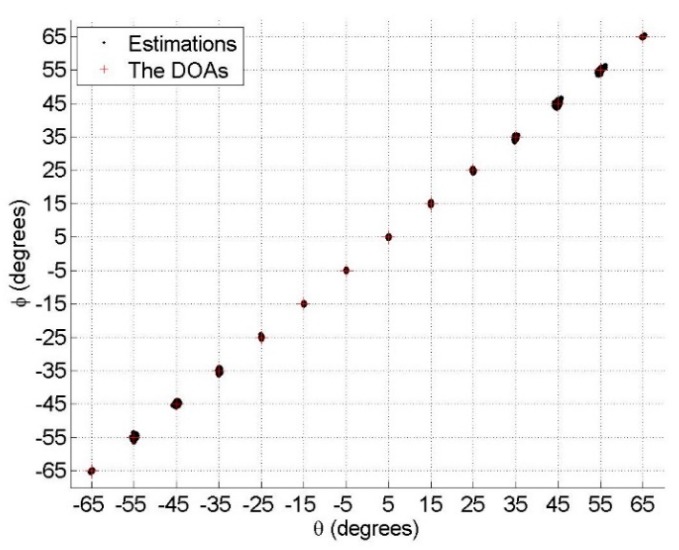
Estimations of 14 sources.

In order to verify that the proposed model can handle 15 DOF, 14 sources with the impinging directions $\theta_{k}=\phi_{k}=-65^{\circ }+(k-1)\times 10^{\circ }$, polarization parameters $\gamma_{k}=30^{{}^{\circ}}$ and $\eta_{k}=30^{{}^{\circ}}$, for k=1,…,14 are taken into consideration. The signal-to-noise ratio (SNR) and the number of snapshots are set to 30 dB and 1000, respectively. The simulation results obtained via 100 Monte Carlo trials are shown in Figure 2. It can be seen from Figure 2 that the proposed model can effectively handle the 14 sources, which cannot be done by both the model in [19] and the benchmark.

### 6.2. Resolution Performance

**Figure 3:**
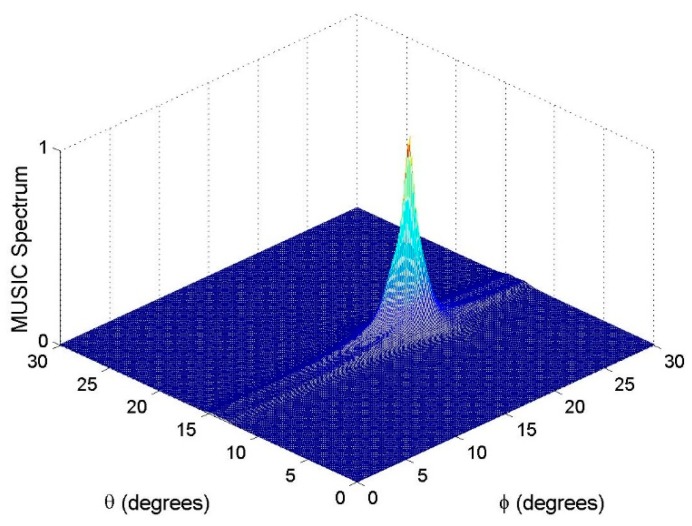
MUSIC spectrum of the method in [19].

**Figure 4:**
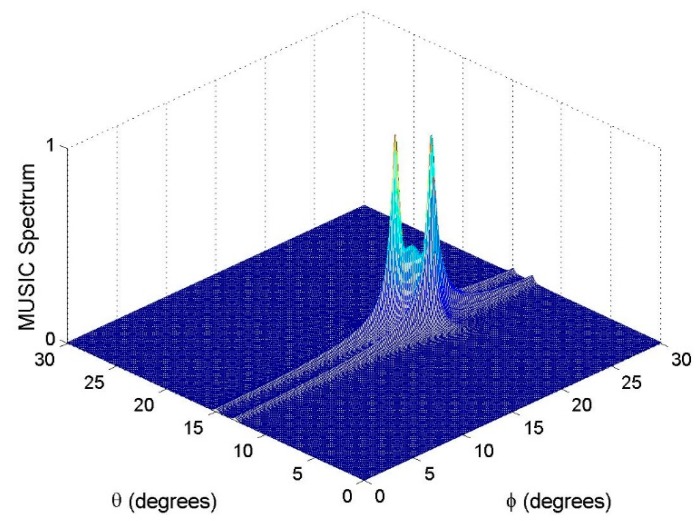
MUSIC spectrum of the benchmark.

**Figure 5:**
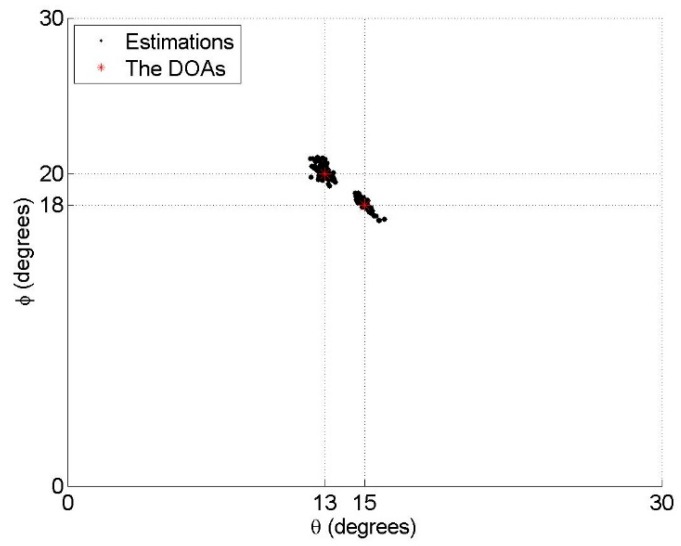
Estimation results of the proposed method, where 100 Monte Carlo trials are carried out.

Assume that there are two close sources in a surveillance region. The two sources with the 2D DOAs as $\left(\theta_{1},\phi_{1})=(15^{{}^{\circ}},18^{{}^{\circ}}\right)$ and $\left(\theta_{2},\phi_{2})=(13^{{}^{\circ}},20^{{}^{\circ}}\right)$ and polarization parameters as $\left(\gamma_{1},\eta_{1})=(\gamma_{2},\eta_{2})=(30^{{}^{\circ}},30^{{}^{\circ}}\right)$ impinge on our nested vector-sensor array. For the purpose of intuitive demonstration, the polarization parameters are assumed to be known. The estimation results with the SNR = 20 dB and *T* = 500 are shown in Figure 3, Figure 4 and Figure 5, respectively. From these figures, one can see that the method in [19] cannot distinguish the two close sources, while both the proposed method and the benchmark can resolve them. It must be emphasized that 12 physical vector sensors are used by the benchmark, whereas six are used by the proposed method.

### 6.3. RMSE vs. SNR and Snapshots

**Figure 6:**
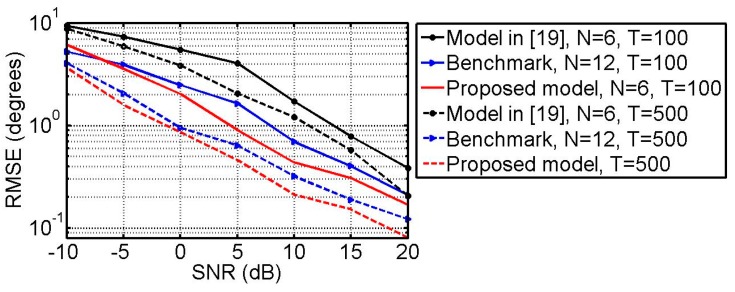
RMSE of the DOA estimates versus SNR with *T* = 100 and *T* = 500.

**Figure 7:**
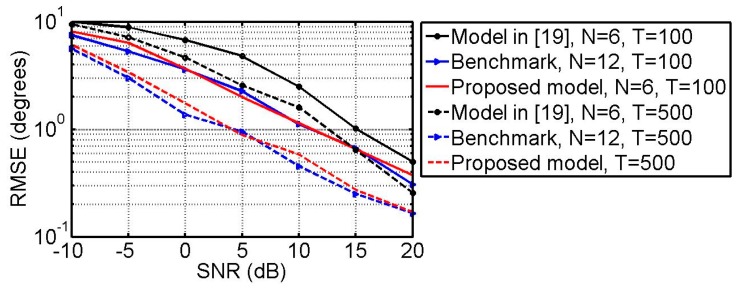
RMSE of the polarization parameter estimates versus SNR with *T* = 100 and *T* = 500.

A source with the impinging direction $\left(\theta ,\phi )=(37^{{}^{\circ}},138^{{}^{\circ}}\right)$ and polarization parameters $\left(\gamma ,\;\eta )=(42^{{}^{\circ}},\;25^{{}^{\circ}}\right)$ is taken as an estimated example. Figure 6 plots the RMSE of the DOA estimates as the function of SNR with T=500 and T=100. Figure 7 plots the RMSE of the polarization parameter estimates versus the SNR with T=500 and T=100. From Figure 6 and Figure 7, one can find that in all cases the proposed model with *N* = 6 outperforms the one in [19] and is very close to the benchmark with *N* = 12.

### 6.4. Detection Performance

**Figure 8:**
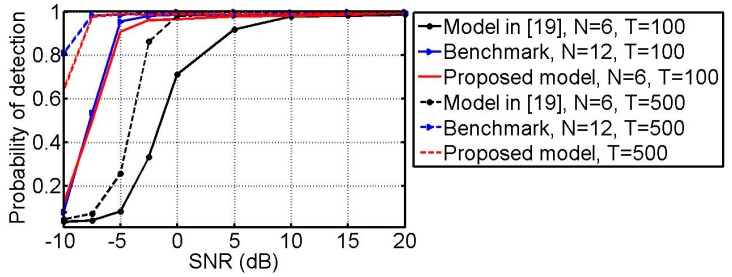
Probability of detection versus SNR with *T* = 100 and *T* = 500.

In the following simulation, the source number detection performance of the models is investigated. Two sources with $\left(\theta_{1},\phi_{1})=(10^{{}^{\circ}},17^{{}^{\circ}}\right)$, $\left(\theta_{2},\phi_{2})=(16^{{}^{\circ}},32^{{}^{\circ}}\right)$, and $\left(\gamma_{k},\eta_{k})=(30^{{}^{\circ}},30^{{}^{\circ}}\right)$ (k=1,2) are taken into consideration. The detection accuracy is defined as $F_{K}/F$, where F is the trial number, and $F_{K}$ is the number of times when the detected source is true. In this example, F=1000. The probability of detection versus SNR with T=100 and T=500 are plotted in Figure 8. One can see from Figure 8 that the detection performance is improved when the SNR and snapshots are increased. In addition, it can also be seen that the proposed method considerably outperforms the one in [19] and performs very close to the benchmark.

### 6.5 RMSE vs. N

**Figure 9:**
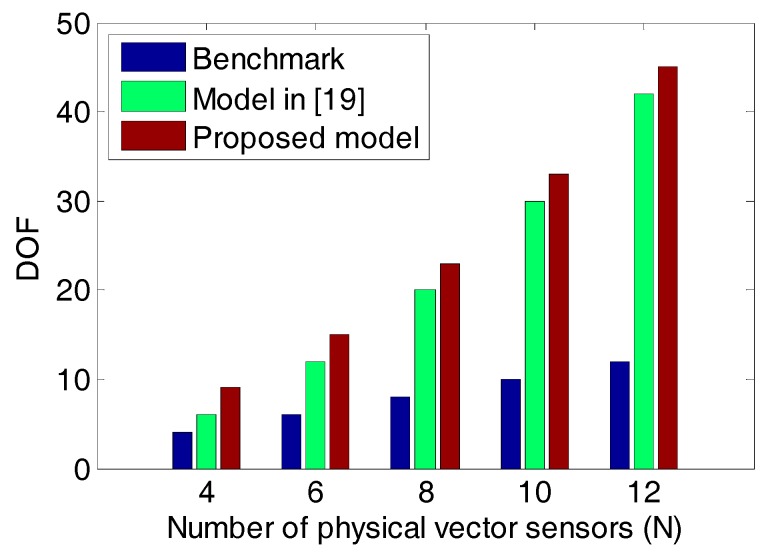
DOF vs. *N*.

**Figure 10:**
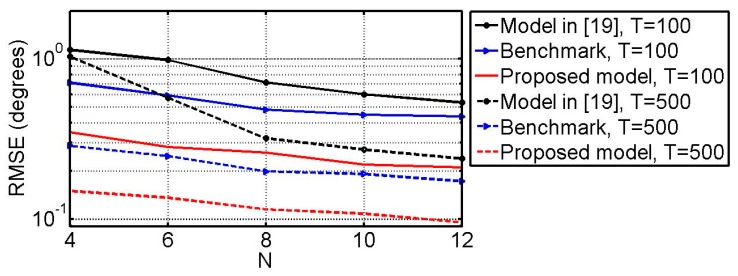
RMSE of the DOA estimates vs. *N*.

**Figure 11:**
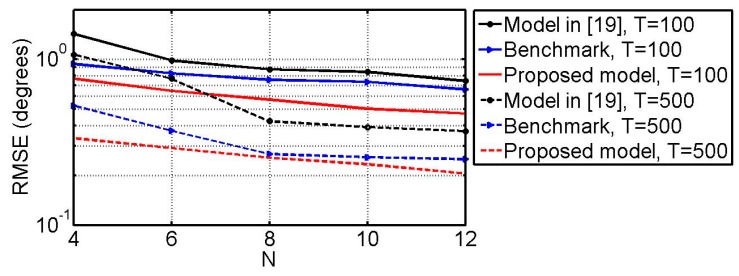
RMSE of the polarization parameter estimates vs. *N*.

What follows, we will compare the three methods by studying the relationships between the RMSEs and the number of physical vector sensors (i.e., *N*). The simulation conditions are the same as the example 6.3 except that SNR = 15 dB and *N* ranges from 4 to 12. The DOFs of the three models versus *N* are shown in Figure 9. Figure 10 shows the RMSEs of the angle estimates versus *N*. Figure 11 gives the RMSEs of the polarization parameter estimates versus *N*. From Figure 9, Figure 10 and Figure 11, one can find that in all cases the method in [19] can provide more DOF than the benchmark while the estimation performance of the benchmark is better than that of the method in [19]. It can also be seen that the proposed method surpasses both of them on the DOF and estimation performance.

### 6.6. Runtime vs. N

**Figure 12:**
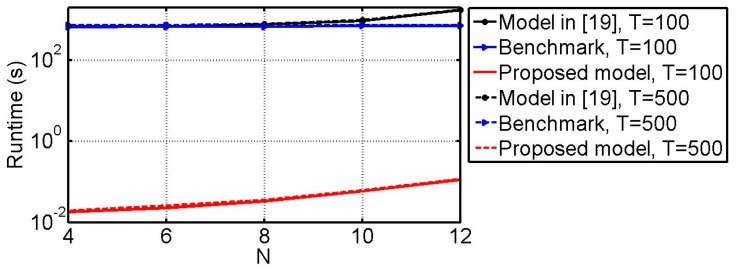
Runtime versus *N*.

As mentioned in [24,26], the computation complexity of the PARAFAC decomposition–based method involves many iterations and largely depends on the received data. Hence, the CPU running time of the proposed method is provided as a reference in this example. All of the compared methods here are implemented in MATLAB R2011a using a PC with Inter(R) Core(TM) i7-6500U CPU @2.50 GHz and 8 G RAM. The simulation conditions are the same as those of example 6.5. All of the simulation results are obtained via 100 Monte Carlo trials. Note that the angular resolution of the benchmark and the method in [19] is fixed to $0.01^{{}^{\circ}}$. The running time for all of the compared methods with respect to *N* is presented in Figure 12. From Figure 12, we can see that for each method the number of snapshots (i.e., *T*) does not have much affect on the running time. It can also be seen that the proposed method takes the least time for all of the cases.

## 7. Conclusions

In this paper, a novel PARAFAC model for processing the nested array with *N* vector sensors, each of which contains *M* components, has been proposed. By dividing the nested vector-sensor array into a series of nested scalar-sensor subarrays and using the autocorrelation matrices of their measurements and cross-correlation matrices among them, the difference co-array of the original nested vector-sensor array is described as a PARAFAC model corresponding to a virtual ULA with $N^{2}/2+N-1$ vector sensors and *M* snapshots. Hence, the proposed model can fully exploit all the measurements of the difference co-array, and allows one using the PARAFAC decomposition to achieve the source localization and polarization estimation efficiently. As demonstrated by simulation results, the proposed model can achieve a better estimate performance efficiently and resolve slightly more sources than the reported ones in the literature.

## Figures and Tables

**Table 1:** The overall procedure of the proposed method.

The Proposed Method
**Input**: Y of the form (5). 1. Extract $Y^{\left(m\right)}$ from Y. 2. Compute R(m,m) and R(m,l), and built $R^{\left(m\right)}$. 3. Compute ${Y^{\prime }}^{\left(m\right)}$ and built $Y^{\prime }$. 4. Extract ${Y^{\prime }}^{\left(m,n_{s}\right)}$ from $Y^{\prime }$ according to (29), and built $Z^{\left(m\right)}$. 5. Compute $Z_{\left\{1\}\{2,3\right\}}^{\left(m\right)}$ and built Z. 6. Obtain ${\hat{\bar{A}}}_{1}$, $\hat{P}$ and $\hat{S}$ from Z. 7. Obtain $\left\{{\hat{\theta }}_{k}\right\}$ from ${\bar{A}}_{1}$. 8. Obtain $\left\{{\hat{\phi }}_{k}\right\}$, $\left\{{\hat{\gamma }}_{k}\right\}$ and $\left\{{\hat{\eta }}_{k}\right\}$ from $\hat{P}$. **Output**: $\left\{{\hat{\theta }}_{k}\right\}$, $\left\{{\hat{\phi }}_{k}\right\}$, $\left\{{\hat{\gamma }}_{k}\right\}$ and $\left\{{\hat{\eta }}_{k}\right\}$.
